# AI achieves board-level performance on the Japan diagnostic radiology board examination through direct image interpretation

**DOI:** 10.1007/s11604-026-01983-x

**Published:** 2026-04-02

**Authors:** Soichiro Miki, Yuichiro Hirano, Takahiro Nakao, Yosuke Yamagishi, Shouhei Hanaoka, Yukihiro Nomura, Akiyoshi Hamada, Noriko Kanemaru, Rintaro Miyo, Masumi Mizuki Takahashi, Reina Hosoi, Takeharu Yoshikawa, Osamu Abe

**Affiliations:** 1 Department of Computational Diagnostic Radiology and Preventive Medicine, The University of Tokyo Hospital, Tokyo, Japan; 2 Division of Radiology and Biomedical Engineering, Graduate School of Medicine, The University of Tokyo, Tokyo, Japan; 3 Department of Radiology, The University of Tokyo Hospital, Tokyo, Japan; 4 Center for Frontier Medical Engineering, Chiba University, Chiba, Japan; 5 Department of Radiology, Nerima Hikarigaoka Hospital, Tokyo, Japan; 6 Department of Radiology, Juntendo University Hospital, Tokyo, Japan

**Keywords:** Artificial intelligence (AI), Large language model (LLM), Google Gemini, OpenAI ChatGPT, Anthropic Claude, Japan Diagnostic Radiology Board Examination (JDRBE).

## Abstract

**Purpose:**

To evaluate text-only versus vision-enabled performance of late-2025 large language models (LLMs) on the Japan Diagnostic Radiology Board Examination (JDRBE) and compare model performance with newly board-certified radiologists.

**Materials and methods:**

Image-based questions from the JDRBE 2021 and 2023–2025 were collected, and ground truth answers were determined by expert consensus. Four commercial multimodal LLMs were evaluated: Gemini 2.5 Pro (March 2025, baseline), Gemini 3 Pro, GPT-5.1, and Claude Opus 4.5 (all released in November 2025). Each question was answered with image input (“vision”) and without images (“text-only”). For the JDRBE 2025, subjective legitimacy of responses was independently rated by two radiologists using a five-point Likert scale, and low-rated responses were further analyzed by error type. Additional analyses on the JDRBE 2025 subset included image-shuffling and multi-run variability assessment (five runs). Model accuracies were also compared with those of five newly board-certified radiologists who passed the JDRBE 2025.

**Results:**

Gemini 3 Pro achieved the highest accuracy among all models, scoring 85.3% (279/327) in the vision condition and significantly outperforming its text-only accuracy (74.3%, *P* < 0.001). Gemini 2.5 Pro and Claude Opus 4.5 also improved with image input, whereas GPT-5.1 did not. For the JDRBE 2025, Gemini 3 Pro in the vision condition received the highest legitimacy ratings, and its accuracy (88%) was above the range observed in a reference group of five newly board-certified radiologists (65%–83%), but hallucination was still the most common error type. Image-shuffling analysis using the 2025 subset showed no performance gain in all models, supporting reliance on visual input. Multi-run variability analysis showed high agreement across runs.

**Conclusion:**

Among late-2025 commercial LLMs, Gemini 3 Pro demonstrated board-level performance on the JDRBE through direct medical image interpretation.

**Secondary abstract:**

The performance of vision-enabled large language models on the Japan Diagnostic Radiology Board Examination was evaluated. Among the models released in November 2025, Gemini 3 Pro demonstrated significant capabilities in direct medical image interpretation, achieving accuracy above that of a reference group of five newly board-certified radiologists.

**Supplementary Information:**

The online version contains supplementary material available at 10.1007/s11604-026-01983-x.

## Introduction

Commercial large language models (LLMs) widely available to the general public have extensive medical knowledge, and their application in the field of radiology has been actively investigated. However, many researchers have pointed out that there is still a large gap between the text-based capabilities of multimodal LLMs and their performance in vision-based medical image interpretation. That is, while LLMs perform well when provided with detailed image findings in text form [1], their performance has been far from satisfactory when interpreting medical images alone [2].

Board examinations have been used by many researchers as benchmarks for evaluating LLM performance, since they can measure problem-solving ability close to real clinical practice [3]. Until 2024, many reports showed that, even when images were provided, multimodal LLMs did not improve their scores on various medical-related examinations [4, 5], including radiology-related examinations [6–9]. Nguyen et al. reported in their meta-analysis that visual input was negatively associated with accuracy when ChatGPT solved radiology board examinations across multiple countries [10]. Later, Gemini 2.5 Pro (Google, Mountain View, CA), released in March 2025, became the first mainstream LLM to significantly improve its scores with image input on the Japan Diagnostic Radiology Board Examination (JDRBE), suggesting that LLMs finally began to directly interpret board-level medical images [11, 12]. Similarly, early-2025 LLMs have demonstrated promising performance on image-based questions from European Diploma in Radiology (EDiR) [13] and Japanese radiological technologist examination [14, 15].

In November 2025, major vendors released their next-generation flagship models: Gemini 3 series by Google [16], GPT-5.1 series by OpenAI (San Francisco, CA) [17], and Claude Opus 4.5 by Anthropic (San Francisco, CA) [18]. Their emphasis on multimodal capabilities varies; while Gemini 3 strongly emphasizes its vision capabilities, Claude Opus 4.5 appears to focus primarily on coding and automation. The performance of this generation of models in radiology has not yet been reported in the literature.

The JDRBE is an examination that evaluates comprehensive knowledge and expertise in diagnostic radiology. Candidates must complete at least five years of radiology training to be eligible. Nearly all questions contain medical images from various modalities, and the questions are not publicly available online, making it a suitable benchmark to assess LLMs’ capability to interpret medical images.

The purpose of this study was to evaluate the performance of the latest multimodal LLMs, also known as vision-language models or VLMs, on the JDRBE. Additionally, we compared their performance with that of newly board-certified radiologists who passed the JDRBE 2025.

## Methods

This was a retrospective study that used data available online to all members of the Japan Radiological Society (JRS). No question contained personally identifiable information, and we used application programming interfaces (APIs) in a way that ensured no data were used for model training. Therefore, approval from the Institutional Review Board was waived.

### Question and answer dataset

We expanded the dataset from our previous study [11] by adding image-based questions and their answers from the JDRBE 2025 held in August 2025. Details are provided in our previous study. Briefly, the question text and images were extracted from PDF files available for download to JRS members. The question text was used as-is without translation into English. The existing dataset contained 233 questions from the JDRBE 2021, 2023, and 2024 (Questions from the JDRBE 2022 were excluded because we failed to extract images from the PDF file).

The official correct answers of the JDRBE are not available online, and were not provided by the JRS upon request. Therefore, the ground truth was determined by consensus among three or more board-certified radiologists, consulting textbooks and online resources as needed. Questions for which no agreement could be reached were excluded from the accuracy calculation. For the JDRBE 2025, the ground truth was determined by three board-certified radiologists: S.M., S.H., and T.Y., with 18, 23, and 30 years of experience in radiology, respectively.

### Models and inference

We evaluated three commercial LLMs, all released in November 2025: Gemini 3 Pro, GPT-5.1, and Claude Opus 4.5. Additionally, based on our previous report [11], we chose Gemini 2.5 Pro (March 2025) as a baseline model for comparison. Although this model fell slightly short of OpenAI o3 in accuracy, it showed the greatest improvement with the addition of images, as well as the highest subjective ratings from radiologists.

Using each vendor’s official API, we submitted each question to each model as a separate request under two conditions: combined text and image input (hereafter, “vision”) and text-only input (“text-only”). Their details, including exact model versions and parameter settings, are summarized in Table 1. For the Claude API, the mandatory *max_tokens* parameter was explicitly set to 4096. Additionally, unlike previous reasoning models from OpenAI, GPT-5.1 was configured not to perform reasoning by default, so we explicitly set the *reasoning.effort* parameter to the previous default value of “medium” to enable reasoning. All other parameters were left at their default values.

**Table 1:** Model specifications and parameter settings

Model	Vendor	Release date	Version	Knowledge cutoff	Specified parameters
Gemini 2.5 Pro	Google	28 Mar 2025	gemini-2.5-pro-preview-03-25	Jan 2025	None (all default)
Gemini 3 Pro	Google	18 Nov 2025	gemini-3-pro-preview	Jan 2025	None (all default)
GPT-5.1	OpenAI	13 Nov 2025	gpt-5.1-2025-11-13	Sep 2024	*reasoning.effort *= medium
Claude Opus 4.5	Anthropic	24 Nov 2025	claude-opus-4-5-20251101	Aug 2025	*max_tokens* = 4096

Responses from Gemini 2.5 Pro for questions from 2024 and earlier were reused from our previous study. Responses for the 2025 questions from this model, as well as all responses from the other models, were generated between November 22 and 27, 2025. Other study settings, including the prompt used for each condition, were identical to those in our previous report. The option selected by the LLM was extracted from the final line of the response using simple string pattern matching. For questions requiring two answers, the response was considered correct only if both correct options were selected.

### Subjective assessment of response quality and error types

To assess the credibility of LLM responses, we conducted a subjective legitimacy analysis based on the JDRBE 2025 subset with ground truths. Two radiologists with different levels of experience (Y.Y., 2 years; T.N., 10 years, board-certified) independently scored the responses from each model. For each response, the two evaluators assigned two ratings using a five-point Likert scale (from 1: very poor to 5: excellent). One was the Findings rating, which evaluated only the ability to identify and describe objective findings in the images, and the other was the Overall rating, which reflected the final impression, including logical reasoning and the validity of the selected answer. The model identity was masked to the evaluators, and the order of responses was randomized.

Subsequently, to clarify error tendencies for each model, the two evaluators re-examined responses with a mean Findings score of 2.5 or below, and classified the reason for the low rating into five predefined types. After independent classification, they determined the final error type through consensus. The error types were as follows: (1) *Image ignored*: no evidence of attempting to interpret the provided images; (2) *Lesion missed*: failure to recognize the key abnormal finding itself; (3) *Misdescription*: the abnormality was recognized but its location or characteristics were inadequately described; (4) *Hallucination*: describing a lesion that does not exist in the image; and (5) *Fundamental error*: critical mistakes such as modality misidentification.

Additionally, as an exploratory subanalysis, we stratified questions on the 2025 subset by imaging modality and anatomic region, and summarized model-wise accuracy and the rate of low-rated responses (mean Findings ≤ 2.5).

### Robustness analysis

To evaluate response stability and reliance on visual input, we conducted two robustness experiments on the JDRBE 2025 subset. First, each question was answered in four additional runs under both vision and text-only conditions to assess output variability. Single-run accuracy (based on the original run) was compared with majority-vote accuracy (ties counted as incorrect). Second, to verify that models actually used the provided images, we created an image-shuffling condition in which each question received images from a different randomly selected question from the same examination, while keeping the question text and answer choices unchanged. Accuracies were compared across the vision, text-only, and shuffled conditions. These experiments were conducted on January 21 and 22, 2026, for all models except the baseline (Gemini 2.5 Pro), which had been discontinued at this point.

### Comparison with human examinees

Since the official passing threshold of the JDRBE is not disclosed, we recruited five newly board-certified radiologists who passed the JDRBE 2025 to reproduce their examination answers. These reproduced answers were then used to estimate the passing threshold and to compare them with the LLM accuracy. They were instructed to reproduce their responses as accurately as possible, relying on memory or on any notes they had taken during the examination. For questions whose answers they did not remember, they thought through them again on their own without using external resources. A custom web form was used to collect the data, and the time to reproduce their answers was neither measured nor restricted. There was a three-month interval between the actual examination (in August) and the reproduction (in November).

### Statistical analysis

McNemar’s exact test was used to compare each model’s accuracy between experimental conditions. For the legitimacy ratings, Friedman’s test was applied first, followed by Wilcoxon signed-rank test with Holm’s correction for post-hoc pairwise comparisons. We also calculated the quadratic weighted kappa between the two raters to assess their agreement. Response variability was summarized using Fleiss’ kappa, standard deviation, and coefficient of variation. Statistical significance was set at *P* < 0.05. All analyses were conducted using Python (version 3.12.6) with the SciPy (version 1.15.2) and Statsmodels (version 0.14.4) libraries.

## Results

**Fig. 1:**
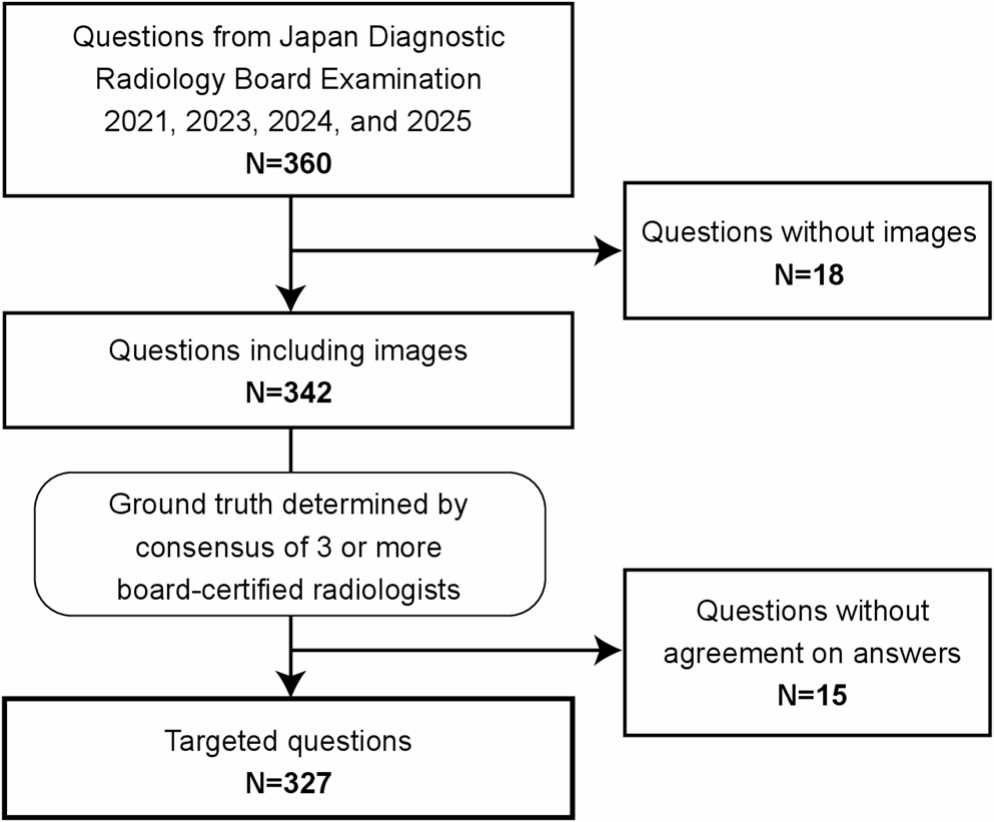
Inclusion criteria for question selection and the number of questions included in this study

For the JDRBE 2025, 96 image-based questions were reviewed, of which two lacked unanimous consensus on the ground truth answer. Therefore, our final dataset with ground truths comprised 327 questions from the JDRBE 2021, 2023, 2024, and 2025 (Fig. 1).

Table 2 shows the number of correct answers for each model under each condition. Among all models, Gemini 3 Pro achieved the highest accuracy in both conditions, scoring 74.3% (243/327) in the text-only condition and 85.3% (279/327) in the vision condition. Gemini 3 Pro, Gemini 2.5 Pro, and Claude Opus 4.5 showed statistically significant improvements in the vision condition compared with the text-only condition, whereas GPT-5.1 did not demonstrate a significant benefit from vision input.

**Table 2:** Number of correct responses out of 327 questions, with (vision) and without (text-only) image input

Model	Text-only	Vision	*P*-value
Gemini 2.5 Pro (baseline)	201 (61.5%)	232 (70.9%)	< 0.001 *
Gemini 3 Pro	243 (74.3%)	279 (85.3%)	< 0.001 *
GPT-5.1	234 (71.6%)	244 (74.6%)	0.237
Claude Opus 4.5	230 (70.3%)	251 (76.8%)	0.005 *

P-values show the significance of differences between the two conditions (McNemar’s exact test, *P < 0.05)

**Fig. 2:**
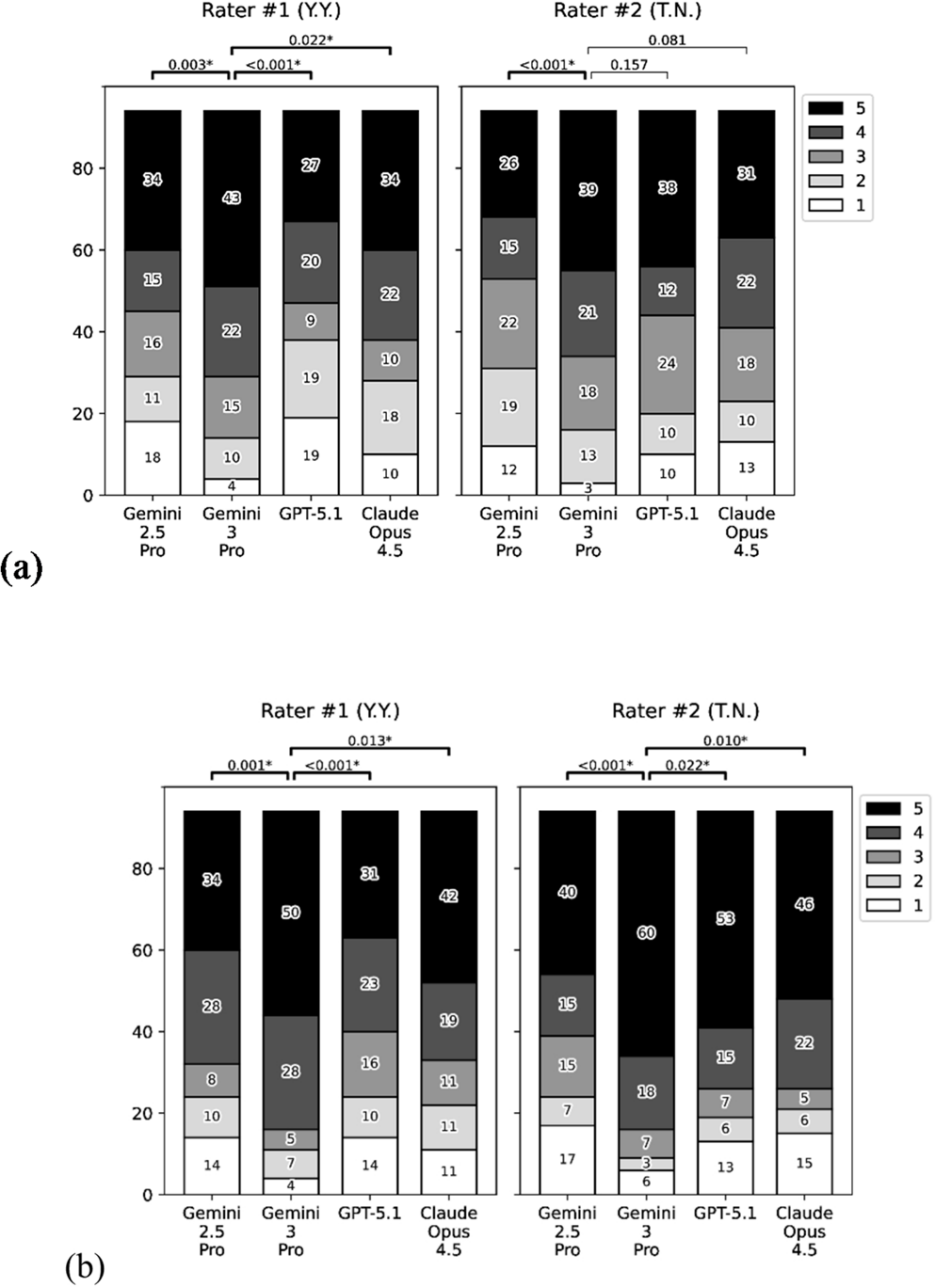
Legitimacy ratings by two independent raters. Stacked bars show the number of responses for each score on a 5-point Likert scale. Numbers above brackets indicate pairwise statistical significance between Gemini 3 Pro and each of the other models (*P < 0.05 after Holm correction). Other model pairs showed no significant differences. (**a**) “Findings” rating, reflecting the model’s ability to identify and describe objective findings in the given images. (**b**) “Overall” rating, reflecting the model’s overall impression of the images

The following results are based on the 94 questions with ground truth in the JDRBE 2025. Figure 2 shows the distribution of the legitimacy scores for each rater and evaluation criterion. The quadratic weighted kappa between the two evaluators was 0.65 for the Findings rating and 0.77 for the Overall rating. Gemini 3 Pro received the highest ratings across both criteria, with a median Overall score of 5 (excellent). Gemini 3 Pro’s Overall ratings were significantly higher than those of all other models (adjusted *P* < 0.05) for both evaluators.

Table 3 shows the distribution of error types for low-rated responses (mean Findings ≤ 2.5). No fundamental errors (e.g., modality misidentification) were observed in any model. Among the four error types observed, hallucination was the most common (*n* = 47; 43%), followed by misdescription (*n* = 37; 34%) and lesion missed (*n* = 22; 20%). Image ignored was rare (*n* = 3; 3%) and observed only in GPT-5.1.

**Table 3:** Error type distribution for responses with a mean Findings rating ≤ 2.5 in the JDRBE 2025 dataset

Model	Image ignored	Lesion missed	Misdescription	Hallucination	Total
Gemini 2.5 Pro (baseline)	0	7	13	15	35
Gemini 3 Pro	0	3	3	7	13
GPT-5.1	3	6	15	10	34
Claude Opus 4.5	0	6	6	15	27

Table 4 summarizes the response variability across five independent trials (the original plus four additional trials) using the 2025 subset (*n* = 94). Fleiss’ kappa values exceeded 0.83 for all models and conditions (“almost perfect agreement” according to the Landis & Koch criteria), with Gemini 3 Pro in the vision condition showing the highest consistency (κ = 0.905). Majority voting yielded no additional performance gain (*P* ≥ 0.25 for all models and conditions).

**Table 4:** Variability metrics across five runs (original plus four additional runs) on the JDRBE 2025 subset (*n* = 94)

Model	Condition	Fleiss’ kappa	SD	CV	Complete agreement	Single-run accuracy	Majority-vote accuracy
Gemini 3 Pro	Vision	0.905	1.93	2.3%	85% (80/94)	88% (83/94)	85% (80/94)
Gemini 3 Pro	Text-only	0.883	1.58	2.0%	82% (77/94)	77% (72/94)	80% (75/94)
GPT-5.1	Vision	0.848	1.75	2.2%	78% (73/94)	80% (75/94)	82% (77/94)
GPT-5.1	Text-only	0.877	1.78	2.3%	81% (76/94)	78% (73/94)	76% (71/94)
Claude Opus 4.5	Vision	0.833	1.61	2.1%	76% (71/94)	80% (75/94)	79% (74/94)
Claude Opus 4.5	Text-only	0.858	3.10	3.9%	78% (73/94)	81% (76/94)	80% (75/94)

Single-run accuracy was based on the original run, and ties in majority voting were counted as incorrect. SD: standard deviation of trial accuracy (% points); CV: coefficient of variation of trial accuracy

In the image-shuffling analysis on the same 2025 subset, Gemini 3 Pro achieved 83, 72, and 65 correct answers under the vision, text-only, and image-shuffled conditions, respectively. Likewise, GPT-5.1 scored 75, 73, and 69, and Claude Opus 4.5 scored 75, 76, and 71. Providing mismatched images consistently reduced LLM performance. These findings support that the performance improvement of Gemini 3 Pro in the vision condition was not due to the mere presence of images, but to genuine interpretation of relevant image content.

For the 2025 subset, the accuracies of the five newly board-certified radiologists ranged from 65% to 83% (mean ± SD: 72% ± 7%). In comparison, the accuracies of the LLMs in the vision condition were 88% for Gemini 3 Pro, 80% for GPT-5.1 and Claude Opus 4.5, and 74% for Gemini 2.5 Pro.

Modality- and region-based accuracies for the 2025 subset are shown in Supplementary Table S1. While subgroup sizes were limited, Gemini 3 Pro generally maintained higher accuracy and lower low-rating rates across subgroups.

## Discussion

Late-2025 general-purpose multimodal LLMs newly evaluated in this study showed substantial progress in radiology compared with the previous generation [11, 12]. Under the text-only condition, all three newly evaluated LLMs outperformed Gemini 2.5 Pro, indicating robust progress in medical knowledge and text-based reasoning capabilities.

In contrast, image interpretation capability varied considerably across models. GPT-5.1 still showed no statistically significant benefit from image input. The Claude series showed statistically significant accuracy improvement from images for the first time, but the gain remained modest. Meanwhile, Gemini 3 Pro advanced its vision-based capabilities already observed in its predecessor. Of particular note is the low proportion of unfavorable Findings ratings (1 or 2); this suggests that this model grounded its responses more in the image content rather than producing plausible descriptions inferred from the question text.

The evolution of multimodal capabilities is evident when viewed chronologically. We previously reported that GPT-4 Turbo with Vision (November 2023) achieved only 45% accuracy on the JDRBE, with vision input not only failing to improve accuracy but also worsening radiologists’ subjective ratings due to many elementary errors [6]. Subsequently, Gemini 2.5 Pro (March 2025) demonstrated an emerging ability to interpret medical images, with an accuracy of 70% [11]. In the present study, Gemini 3 Pro not only surpassed this accuracy even under the text-only condition (74%), but also showed 85% accuracy with images, showing the largest gain in our present and previous reports. Furthermore, the results from response variability analysis indicate that the outputs of this generation of LLMs are sufficiently stable. These findings may suggest that some frontier multimodal models have finally reached a stage where direct image input can support clinical diagnosis, eliminating the need for manual translation of image findings into text.

**Fig. 3:**
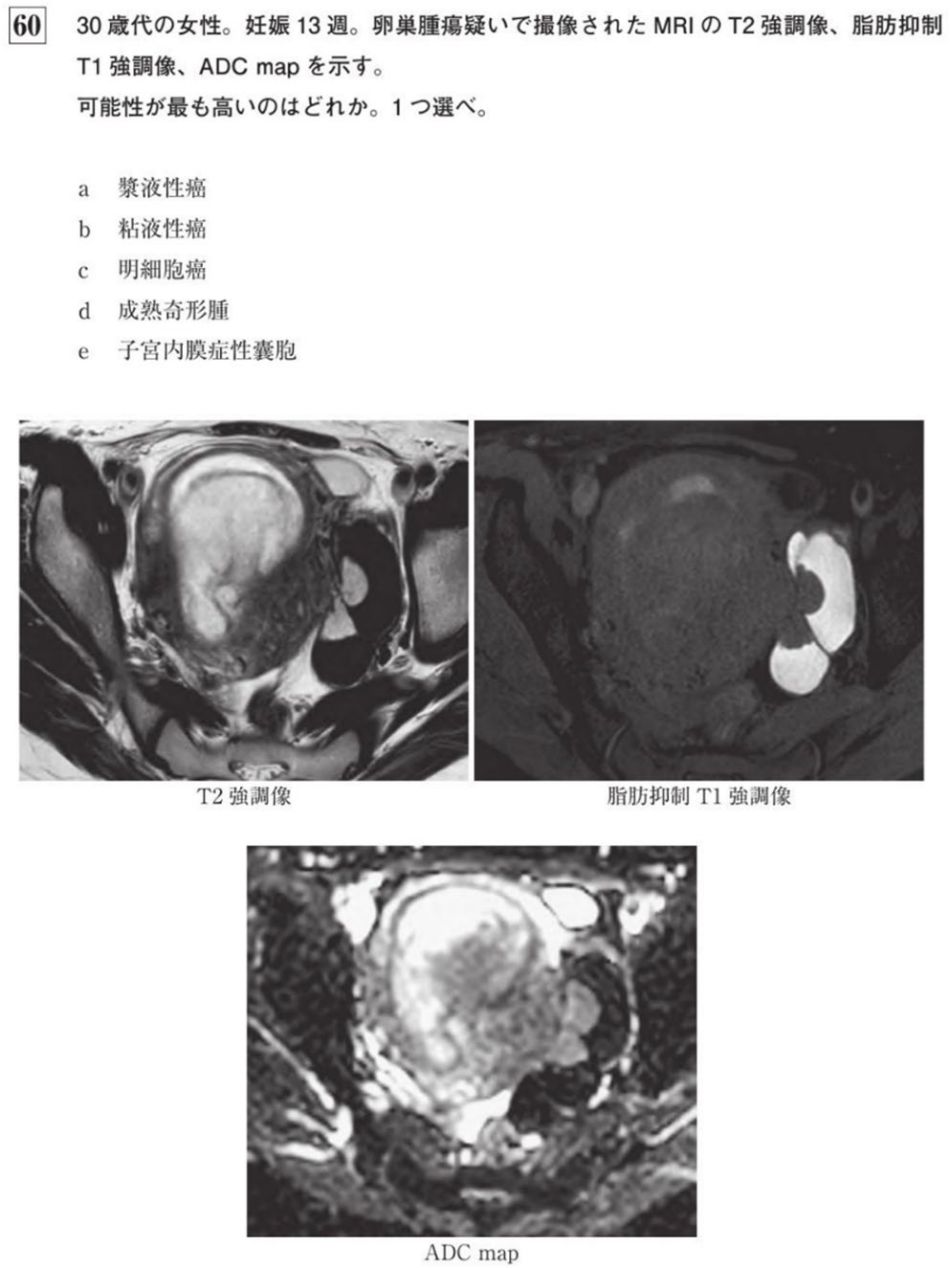
Question 60 from the Japan Diagnostic Radiology Board Examination 2025, reproduced with permission from the Japan Radiological Society. The case involves a pregnant woman in her 30s at 13 weeks of gestation with a suspected ovarian tumor. T2-weighted image, fat-suppressed T1-weighted image, and apparent diffusion coefficient map are shown. The five options are: (a) serous carcinoma, (b) mucinous carcinoma, (c) clear cell carcinoma, (d) mature teratoma, and (e) endometriotic cyst

Figure 3 highlights Gemini 3 Pro’s image interpretation and reasoning capabilities. This case presents a diagnostic challenge involving an evident mural nodule mimicking malignancy within a cystic left ovarian mass. While all four models selected the correct diagnosis of an endometriotic cyst, GPT-5.1 and Claude Opus 4.5 failed to even mention the mural nodule. GPT-5.1 also incorrectly localized the lesion to the right side. Gemini 2.5 Pro noted the nodule but failed to assess its appearance or diffusion characteristics. Only Gemini 3 Pro provided a detailed evaluation of the nodule’s malignant potential. By integrating the smooth margin, absence of restricted diffusion, and the patient’s pregnancy status, it correctly concluded that the lesion was an endometriotic cyst with decidualization and that the likelihood of malignancy was low. In comparison, two out of the five newly board-certified radiologists selected incorrect answers (clear cell carcinoma and mature teratoma).

The reason Gemini 3 Pro outperformed other models in image interpretation remains unclear. Throughout 2025, LLMs made rapid progress in areas such as improved logical reasoning, expanded maximum token limits, and agentic capabilities for autonomous long-running work. However, these areas do not seem directly related to the abilities required for the JDRBE. Google has released medical-specialized open-source LLMs [19], which may have given it an advantage in dataset size over other vendors. However, since Gemini 3 Pro is a closed model, the details have not been disclosed.

Our results do not imply that artificial intelligence (AI) can replace radiologists in the near term. AI is still prone to serious mistakes. Our error type analysis revealed that hallucination was still a common error type for all models, raising concerns about false findings leading to misdiagnosis. For example, in one lung scintigraphy question (images not shown), although both perfusion and ventilation were reduced in a similar pattern, all four models described the ventilation scan as normal, reaching an incorrect diagnosis of a ventilation–perfusion mismatch. While this was a “trick question” that misled most (4 out of 5) human respondents, it demonstrates that AI’s capacity for unbiased image interpretation remains imperfect. Furthermore, in clinical practice, radiologists need to detect important findings among numerous normal images, which is a different skill that was not assessed in our setting.

The JDRBE is a real-world benchmark designed to assess high-level integration of text and image reasoning, making it inherently difficult to isolate pure image interpretation. Our legitimacy analysis evaluated responses along two axes, Findings and Overall, but similar rating patterns across these axes suggested that subjective scoring could not fully disentangle visual reasoning from text-based reasoning. While radiologists can identify most of the plausible descriptions generated solely from textual cues as hallucinations, some may be correct by chance and receive high subjective ratings [20]. Thus, even high subjective ratings do not necessarily confirm that the model actually interpreted the underlying images. Our image-shuffling analysis demonstrates that image content contributed to the improved scores, but it does not prove that the models rely on images to the same extent as humans. On the other hand, simple visual question answering tasks available to the public, such as VQA-RAD [21], cannot adequately capture the complexity of integrated clinical reasoning required in real-world radiology practice. In the future, image-only benchmarks with board-level complexity, such as those proposed by Datta et al. [22], may provide insights that complement our real-world examination data by isolating pure image interpretation ability.

Our estimation of the passing threshold is limited by the small sample size and the three-month interval between the examination and the answer reproduction process. During this period, the participants may have forgotten some answers or acquired new knowledge through review, so our approach does not accurately reflect the passing threshold at that time. Nevertheless, all five examinees passed the examination, and Gemini 3 Pro in the vision condition scored above the highest accuracy among them. Although the small sample size precludes a definitive conclusion, our results suggest that the best-performing model of this generation can pass the JDRBE with image input, and even without images, it is approaching, or may have reached, the passing level. Other researchers have reported that Gemini 2.5 Pro and GPT-5 surpassed the official passing threshold of EDiR [13], and that six models outperformed graduating residents in the American College of Radiology In-Training Exams [23].

In this study, we used a simple prompt across all models and relied on each model’s default parameter settings. In other words, we performed no prompt engineering, parameter tuning, or performance-maximization techniques such as pre-translating questions into English. This approach allows us to evaluate performance fairly based on how general users typically use AI and avoids biases from over-optimization for specific vendors’ models or model generations. However, it should be noted that more aggressive optimization could potentially achieve better performance for individual models than reported here. In particular, some low Findings ratings for GPT-5.1 due to the model’s failure to describe the images could have been resolved through prompt adjustments.

In addition to the ones discussed above, this study has several other limitations. First, the ground truth was determined by expert consensus among several radiologists, and does not have the same validity as official answers. Second, all evaluated systems were proprietary API models whose internal updates are not fully transparent, and reproducibility across time may be limited.

In conclusion, commercial LLMs released in late 2025 showed steady gains in text-based performance, while their vision-based performance was model-dependent. Among the evaluated models, Gemini 3 Pro demonstrated the strongest capability for board-level medical image interpretation.

## Supplementary Information

Below is the link to the electronic supplementary material.

Supplementary Material 1

## Data Availability

Questions of JDRBE are available for download on the member-only section of the JRS website. Data generated in this study, including model responses, legitimacy scores, and ground truth answers, are available from the corresponding author upon reasonable request.
