# Supplementary material for: AI achieves board-level performance on the Japan diagnostic radiology board examination through direct image interpretation
Source: Jpn J Radiol. 2026 Apr 2;44(8):1492–9. doi: 10.1007/s11604-026-01983-x (PMC13400479; doi:10.1007/s11604-026-01983-x)
Supplement: Supplementary file 1 — Supplementary Material 1 [file 11604_2026_1983_MOESM1_ESM.pdf]

Supplementary Table S1

Stratified analysis by modality and anatomic region for the JDRBE 2025 dataset with ground-truth answers (n = 94). The summed modality counts exceed 94 because some questions involved multiple modalities. Classification was conducted by a board-certified diagnostic radiologist (S.M., 18 years of experience). CT: computed tomography; MR: magnetic resonance; NM: nuclear medicine; XR: X-ray; IR: interventional radiology; US: ultrasound.

a) Accuracy by modality and model (and five human participants)

Modality	Gemini 2.5 Pro	Gemini 3 Pro	GPT-5.1	Claude Opus 4.5	Human
CT (n=36)	27/36 (75%)	34/36 (94%)	28/36 (78%)	30/36 (83%)	133/180 (74%)
MR (n=31)	24/31 (77%)	26/31 (84%)	26/31 (84%)	24/31 (77%)	116/155 (75%)
NM (n=23)	16/23 (70%)	20/23 (87%)	17/23 (74%)	19/23 (83%)	84/115 (73%)
XR (n=10)	7/10 (70%)	7/10 (70%)	7/10 (70%)	6/10 (60%)	23/50 (46%)
IR (n=7)	6/7 (86%)	7/7 (100%)	7/7 (100%)	7/7 (100%)	25/35 (71%)
US (n=1)	1/1 (100%)	1/1 (100%)	1/1 (100%)	1/1 (100%)	4/5 (80%)

b) Low-rating rate (mean Findings ≤ 2.5) by modality and model

Modality	Gemini 2.5 Pro	Gemini 3 Pro	GPT-5.1	Claude Opus 4.5
CT (n=36)	16/36 (44%)	6/36 (17%)	18/36 (50%)	11/36 (31%)
MR (n=31)	10/31 (32%)	3/31 (10%)	10/31 (32%)	6/31 (19%)
NM (n=23)	8/23 (35%)	4/23 (17%)	8/23 (35%)	8/23 (35%)
XR (n=10)	2/10 (20%)	1/10 (10%)	3/10 (30%)	3/10 (30%)
IR (n=7)	1/7 (14%)	0/7 (0%)	1/7 (14%)	0/7 (0%)
US (n=1)	0/1 (0%)	0/1 (0%)	0/1 (0%)	0/1 (0%)

c) Accuracy by anatomic region and model (and five human participants)

Anatomic region	Gemini 2.5 Pro	Gemini 3 Pro	GPT-5.1	Claude Opus 4.5	Human
Head & Neck (n=21)	18/21 (86%)	19/21 (90%)	18/21 (86%)	19/21 (90%)	79/105 (75%)
Chest (n=31)	21/31 (68%)	26/31 (84%)	20/31 (65%)	21/31 (68%)	113/155 (73%)
Abdomen (n=33)	25/33 (76%)	31/33 (94%)	30/33 (91%)	28/33 (85%)	114/165 (69%)
Musculoskeletal (n=9)	6/9 (67%)	7/9 (78%)	7/9 (78%)	7/9 (78%)	31/45 (69%)

d) Low-rating rate (mean Findings ≤ 2.5) by anatomic region and model

Anatomic region	Gemini 2.5 Pro	Gemini 3 Pro	GPT-5.1	Claude Opus 4.5
Head & Neck (n=21)	5/21 (24%)	3/21 (14%)	6/21 (29%)	5/21 (24%)
Chest (n=31)	17/31 (55%)	7/31 (23%)	16/31 (52%)	13/31 (42%)
Abdomen (n=33)	11/33 (33%)	2/33 (6%)	9/33 (27%)	8/33 (24%)
Musculoskeletal (n=9)	2/9 (22%)	1/9 (11%)	3/9 (33%)	1/9 (11%)
